# Evaluating laboratory request forms submitted to haematology and blood transfusion departments at a hospital in Northwest Nigeria

**DOI:** 10.4102/ajlm.v5i1.381

**Published:** 2016-05-12

**Authors:** Feyisayo Jegede, Henry A. Mbah, Ado Dakata, Dalhatu H. Gwarzo, Surajudeen A. Abdulrahman, Aisha Kuliya-Gwarzo

**Affiliations:** 1 Family Health International 360 (FHI360), Department of Laboratory Services, Abuja, Nigeria; 2 LabTrail Global, Smyrna, Delaware, United States; 3 Department of Haematology, Bayero University/Aminu Kano Teaching Hospital, Kano, Nigeria

## Abstract

**Background:**

The laboratory request form (LRF) is a communication link between laboratories, requesting physicians and users of laboratory services. Inadequate information or errors arising from the process of filling out LRFs can significantly impact the quality of laboratory results and, ultimately, patient outcomes.

**Objective:**

We assessed routinely-submitted LRFs to determine the degree of correctness, completeness and consistency.

**Methods:**

LRFs submitted to the Department of Haematology (DH) and Blood Transfusion Services (BTS) of Aminu Kano Teaching Hospital in Kano, Nigeria, between October 2014 and December 2014, were evaluated for completion of all items on the forms. Performance in four quality indicator domains, including patient identifiers, test request details, laboratory details and physician details, was derived as a composite percentage.

**Results:**

Of the 2084 LRFs evaluated, 999 were from DH and 1085 from BTS. Overall, LRF completeness was 89.5% for DH and 81.2% for BTS. Information on patient name, patient location and laboratory number were 100% complete for DH, whereas only patient name was 100% complete for BTS. Incomplete information was mostly encountered on BTS forms for physician’s signature (60.8%) and signature of laboratory receiver (63.5%). None of the DH and only 9.4% of BTS LRFs met all quality indicator indices.

**Conclusion:**

The level of completion of LRFs from these two departments was suboptimal. This underscores the need to review and redesign the LRF, improve on training and communication between laboratory and clinical staff and review specimen rejection practices.

## Introduction

Efficient laboratory service remains a foundation of modern healthcare systems. Laboratory testing is an essential part of the clinical decision-making process, because it provides the majority of critical information required for making timely and informed decisions for patient care.^1^ Relationships with laboratories and utilisation of laboratory services by physicians and other stakeholders in the healthcare system occur mainly through the use of laboratory request forms (LRFs) for two-way communication. Requesting physicians may not fully utilize this important communication medium.^2^ Inadequate information or errors arising from the process of filling out LRFs can have a significant impact on the quality of laboratory outputs and, ultimately, on patient safety.^3,4^

The notion of the brain-to-brain loop for laboratory diagnostics was first introduced by George Lundberg over 30 years ago.^5^ The first step in this loop model involves the selection of appropriate laboratory tests in the brain of the physician, which is then communicated through the LRF. This is followed by numerous intermediary steps, such as identification of the patient, specimen collection and specimen handling; and then by the actual specimen analysis in the laboratory. The last steps involve the release of test results, either manually or electronically, for the physician’s review and reaction to the laboratory information, the interpretation of the results and the implementation of appropriate clinical action.^5^ Traditionally, laboratory practice is divided into three phases (pre-analytical, analytical and post-analytical).^6^ Evidence shows that the majority of laboratory errors (50% – 70%) occur during the pre-analytical phase and involve the handling of the LRF.^7^ Errors occurring during the analytical phase average less than 10%,^8^ whereas errors occurring during the the post-analytical phase average about 15%.^3^ The pre- and post-analytical phases lie outside of the control of the laboratory, but contribute approximately 93% of total laboratory errors across the entire testing process.^9,10,11,12^

The most frequent pre-analytical errors as compiled by Lippi^12^ are: missing sample and/or test request; wrong/missing identification; *in vitro* haemolysis; undue clotting; wrong container; and contamination from infusion route. Other errors include: insufficient sample; inappropriate blood-to-anticoagulant ratio; insufficient mixing of the sample; or inappropriate transport and storage conditions. Insufficient information or omission of information on the LRF may lead to laboratory errors,^13^ as well as make result interpretation difficult and delay communications with the requesting physician, moreso in patients with life-threatening medical conditions. Misidentification of either the patient or the requested test have also been encountered frequently.^14^ The LRF not only provides information about the laboratory test being requested, but is also used to communicate results back to physicians and patients. The standard LRF contains demographic data and other information, such as location of the patient, laboratory information, physician’s name and signature, telephone number of the requesting physician, amongst others. Pre-analytical errors, such as the absence of important clinical information on LRF, can have serious effects on patient care by causing post-analytical errors, such as inappropriate interpretative comments.^15^ The majority of errors occurring during the pre-analytical phase are a result of individual or system design defects.^16^ The pre-analytical phase should be prioritised so as to reduce errors across the entire laboratory testing process.^16^ In Australia, planned interventions and sustained improvements in compliance with standards resulted in an immediate reduction in the proportion of incomplete LRFs, from 43% to 2%.^14^

In developed countries, laboratory quality management systems have been institutionalised, with functional and robust monitoring systems in place to detect and minimise errors before they occur at any phase in the laboratory work flow. Unfortunately, the converse is true in most laboratories in developing countries, such as Nigeria.^17,18^ In these countries, the focus tends to be on the analytical phase of the work flow without consideration of other factors or variables beyond the control of the laboratory. In Nigeria, there are few studies on the handling of LRFs or the impact of the LRF on the pre-analytical phase of laboratory process. The objective of this study was to assess routinely-submitted LRFs for correctness, completeness and consistency and to evaluate the contributions of the LRF to quality service delivery.

## Research method and design

### Ethical considerations

The study protocol was reviewed and approved by the Aminu Kano Teaching Hospital ethical committee (reference number: AKTH/MAC/SUB/12A-3/VI/1330) in line with the international standard of research requirement. Team members were trained to retain patient confidentiality and patient names were not collected as part of the data set.

### Study design and setting

This was a retrospective, cross-sectional, descriptive study. All LRFs submitted to the Department of Haematology (DH) and the Blood Transfusion Services (BTS) of Aminu Kano Teaching Hospital, Kano, Nigeria, from October 2014 to December 2014 were reviewed systematically and evaluated for completeness, correctness and consistency. Selection of DH and BTS LRFs was based on presumed availability of LRFs and the high workload of these departments. Aminu Kano Teaching Hospital is a tertiary health institution located in Kano State in northwestern Nigeria. It is a 600-bed hospital which serves as the referral centre to Kano and other neighbouring states, including Katsina, Jigawa and Bauchi.

### Data collection and analysis

Hard copies of both inpatient and outpatient LRFs received for routine laboratory investigations were reviewed and evaluated for the purposes of this study. Data from the DH and BTS were extracted manually from the LRFs and entered into an Excel file (Microsoft, Redmond, Washington, United States), then collated, cleaned and reviewed before analysis using the Statistical Package for Social Scientists (SPSS; version 21/2012; IBM, Armonk, New York, United States). A score of 1 was used to indicate complete and correctly-filled information, whereas a score of 0 was recorded when any item was missing.

A frequency distribution table was created to summarise the data collected. Data were analysed and categorised into groups of quality indicators (QI), based on International Federation of Clinical Chemistry-Working Group (IFCC-WG) guidelines.^19^ The QIs used were: (1) patient identifiers (name, age, sex and unit number); (2) appropriateness of test request (request date and specimen type); (3) availability and completeness of laboratory details (eg. laboratory number and reference range); and (4) availability and completeness of physician’s details (doctor’s name and signature, name of consultant, phone and fax number). In this setting, ‘consultants’ head a medical team and are the most experienced senior clinicians.

## Results

A total of 2084 LRFs were evaluated, comprising 999 from DH and 1085 from BTS. DH forms requested a total of 12 data elements, whereas BTS forms requested a total of 18 data elements.

### Department of Haematology

Overall, 89.5% of DH forms were filled out completely (Table 1). Of all the required information on the LRF, only patient name, location within the hospital (ward) and laboratory number were filled out both completely and correctly for all patients. Patient age, sex, request date, unit number, specimen type and clinical information were both available and correctly filled out for over 98% of the forms. Of the 244 (24.4%) LRFs with incomplete information for either the doctor’s name and signature or the consultant’s name, a greater number of forms (*n* = 145; 14.5%) were missing the consultant’s name as compared with those missing the physician’s name and signature (*n* = 99; 9.9%). The current DH LRF does not request the phone number of the requesting physician, the time of specimen collection, the signature of the laboratory supervisor/manager to validate the patient results or the reference range.

**TABLE 1:** Completeness of laboratory request forms submitted to Department of Haematology of Aminu Kano Teaching Hospital, Kano, Nigeria, from October 2014 to December 2014 (*n* = 999).

Data element	Completed *n* (%)	Not completed *n* (%)
Patient’s full name	999 (100.0)	0 (0.0)
Age (years)	980 (98.1)	19 (1.9)
Sex (male or female)	987 (98.8)	12 (1.2)
Request date	994 (99.5)	5 (0.5)
Patient’s location (ward)	999 (100.0)	0 (0.0)
Unit number/Hospital number	985 (98.6)	14 (1.4)
Specimen type	996 (99.7)	3 (0.3)
Signature of specimen receiver	43 (4.3)	956 (95.7)
Physician’s name and signature	900 (90.1)	99 (9.9)
Consultant’s name	854 (85.5)	145 (14.5)
Clinical information/diagnosis	997 (99.8)	2 (0.2)
Laboratory number	999 (100.0)	0 (0.0)

**Overall**	**(89.5)**	**(10.5)**

### Blood transfusion service

Overall, 81.2% of BTS LRFs were filled out completely (Table 2), which was lower than for the DH. Of all the variables expected to be completed on the LRFs, only patient name was available and completed on all forms examined. Conversely, none of the BTS forms had time of request completed. As observed with the DH forms, patient age was completed on 1071 forms (98.8%) and sex was completed on 1056 forms (97.4%). For 62 forms (5.7%), hospital/unit number was not indicated. For test request information, clinical information/diagnosis was completed on 877 forms (80.9%), whereas 1000 forms (92.2%) had information on specimen type completed. Similarly, date of request was completed on 1005 forms (92.7%). For other LRF details, number of units of blood requested was complete on 1013 forms (93.4%), type of product requested on 866 forms (79.8%), and degree of urgency on 811 forms (74.7%). The physician’s name was missing on 124 forms (11.4%) and the physician’s signature on 660 forms (60.8%), whereas 271 forms (25%) were missing the supervising consultant’s name. All but one (0.1%) of the BTS forms had laboratory number completed. Almost all forms (*n* = 1082; 99.7%) had the date of sample collection completed, whereas only 396 (36.5%) had the signature of the laboratory receptionist (receiver) completed. The current BTS form does not request the phone number of the requesting physician or the time of the request.

**TABLE 2:** Completeness of laboratory request forms submitted to Blood Transfusion Services of Aminu Kano Teaching Hospital, Kano, Nigeria, from October 2014 to December 2014 (*n* = 1085).

Data element	Completed *n* (%)	Not completed *n* (%)
Patient’s full name	1085 (100.0)	0 (0.0)
Age (years)	1071 (98.8)	14 (1.2)
Sex	1056 (97.4)	29 (2.6)
Unit number/Hospital number	1023 (94.3)	62 (5.7)
Laboratory request available	1083 (99.8)	2 (0.2)
Clinical information/diagnosis	877 (80.9)	208 (19.1)
Consultant’s name	814 (75.0)	271 (25.0)
Requesting physician’s full name	961 (88.6)	124 (11.4)
Date of request	1005 (92.7)	80 (7.3)
Physician’s signature	425 (39.2)	660 (60.8)
Patient’s location (ward)	1081 (99.6)	4 (0.4)
Specimen type	1000 (92.2)	85 (7.8)
Laboratory number	1084 (99.9)	1 (0.1)
Date of sample collection	1082 (99.7)	3 (0.3)
Signature of laboratory receiver	396 (36.5)	689 (63.5)
Number of units requested	1013 (93.4)	72 (6.6)
Type of product requested	866 (79.8)	219 (20.2)
Degree of urgency	811 (74.7)	274 (25.3)

**Overall**	**(81.2)**	(18.8)

### Major quality indicators

Overall, the most frequently occurring data quality gap identified on DH forms was completion of laboratory details (*n* = 0), followed by physician’s details, which were complete on 762 of the forms examined (76.3%; Table 3). Patient identifiers were available and complete on 968 DH forms (96.9%), and 991 forms (99.2%) had relevant fields for test request completed. The most frequently observed quality gap on BTS forms was completion of physician’s details (*n* = 349; 32.2%), followed by completion of laboratory details (*n* = 396; 36.5%) and test request details (*n* = 522; 48.1%). The least commonly occurring data quality gap on BTS forms was completion of patient identifiers (*n* = 1043; 96.1%). None of the DH request forms and only 102 BTS forms (9.4%) examined met all of the QIs analysed in this study. The majority of BTS forms (*n* = 983; 90.6%) met one or more QI requirements.

**TABLE 3:** Completeness of laboratory request forms submitted to Department of Haematology (*n* = 999) and Blood Transfusion Services (*n* = 1085) of Aminu Kano Teaching Hospital, Kano, Nigeria, from October 2014 to December 2014: Analysis based on major quality indicators according to International Federation of Clinical Chemistry - Working Group.^19^

Quality Indicators	Completed DH LRFs *n* (%)	Completed BTS LRFs *n* (%)
Patient identifiers	968 (96.9)	1043 (96.1)
Test request details	991 (99.2)	522 (48.1)
Physician’s details	762 (76.3)	349 (32.2)
Laboratory details	0 (0.0)	396 (36.5)
Met all quality indicators	0 (0.0)	102 (9.4)

DH, Department of Haematology; LRF, laboratory request form; BTS, Blood Transfusion Services.

## Discussion

The study revealed that of the 12 required pieces of information on LRFs from the DH, only three (patient’s name, location within the hospital [ward] and laboratory number) were filled out both completely and correctly for all patients. For LRFs from the BTS, of 18 pieces of required information, only patient name was filled out both completely and correctly for all patients. The most commonly incomplete item on DH forms was the specimen receiver’s signature, whereas for BTS forms, specimen receiver’s signature and doctor’s signature were commonly incomplete.

This study demonstrated that a higher proportion of LRFs from the DH were completed compared with LRFs from the BTS (89.5% for DH vs 81.2% for BTS). These findings are in agreement with a proportion of 84% completion previously reported from a similar study conducted in Ile-Ife, Nigeria.^20^ However, our finding is in sharp contrast with a proportion of 1.73% for LRF completion reported from a similar study conducted in Lagos, Nigeria.^21^

In our study, the name of the requesting physician was completed on most DH and BTS forms (90.1% for DH; 88.6% for BTS). Unfortunately, because of the design of the LRF, the contact details of the requesting physician, which may be needed for follow-up, are not requested. Various studies conducted in South Africa have reported comparable (89.6%)^22^ or a lower (65.2%)^15^ proportions for missing physician details and contact information. Consultant name was well documented, with complete information on 75% of BTS forms and 85.5% of DH forms. However, these proportions are lower than an Ile-Ife study reporting 96.6% completion of consultant-in-charge information.^20^ An Australian study reported that 43% of forms lacked complete information; missing items included physician’s name and pager number(s).^14^ One reason for this variation in our setting may be attributed to work pressure on junior physicians and improper orientation regarding the impact of incomplete LRFs on the quality of patient care. This training is done by senior physicians without collaboration with laboratory professionals. In Nigeria, it is not uncommon for physicians to be reluctant to follow guidance from medical laboratory professionals because of the prevailing power differential between physicians and other health care providers.^23^ Healthcare workers’ attitudes^18,24^ toward the completion of LRFs cannot be overlooked, following reports of poor documentation of laboratory processes. In Nigeria, it is common for staff to consider such documentation as unnecessary paperwork and an extra burden.^18,24^

Our study reported that 99.8% of DH forms and 80.9% of BTS forms had clinical diagnosis details completed. This is consistent with a similar study conducted in Ile-Ife, Nigeria, which reported 93.2% completion of clinical diagnosis details.^20^ However, our findings contrast with the 65.9% completion of clinical details reported in Lagos, Nigeria,^21^ 77% completion reported at Nepal University Teaching Hospital^25^ and 22.7% completion reported at Ghana Tertiary Hospital.^13^ Furthermore, a study of LRFs conducted in Cape Town, South Africa, reported that 20.8% had no diagnosis information and 25.3% had diagnosis information given in an abbreviated form.^15^ In an Indian study, diagnosis was not indicated on 61.20% of forms.^26^ Unfortunately in these cases, critical results found by the laboratory for 17.30% of the patients could not be communicated to them by the physicians because of incomplete forms.^26^

In our study, about 98% of DH and BTS forms had complete information for patient age and sex, which is comparable to 86.4% completion for age and 99.8% completion for sex reported from the Ile-Ife, Nigeria study.^20^ Our findings are higher than the Lagos, Nigeria study, which reported 68% completion for patient age,^21^ as well as the much lower completion reported for patient age (25.6%) and sex (32.7%) in a Ghanaian study.^13^ Both our report and the Ile-Ife study^20^ found that the only well-completed parameter on the LRFs was patient information. The design of the request form may itself be a contributing factor to eliciting completion of some desired information, as patient demographic characteristics are displayed prominently at the beginning of each form. However, in addition to patient information, the Lagos study found that the referring physician’s name was the most completed information (99%),^21^ which is better than our findings of 90.1% for DH forms and 88.6% for BTS forms.

We found that documentation of specimen type was better for DH (99.7%) compared with BTS (92.2%). This is close to the 89.9% reported in the Ile-Ife, Nigeria study.^20^ However, our finding contrasts with the much lower ~12% reported at the North Indian Neuropsychiatry Institute.^26^

It is worth noting that only 4.3% of DH forms appropriately captured the signature of the laboratory receptionist. Reception of samples from inpatients is usually in bulk; as such, the receptionist may be overwhelmed with work and therefore not able to individually sign all LRFs. Other contributing factors may be: lack of proper training; and commitment to utilise standard operating procedures and guidelines in the laboratory. More importantly, information on result verification by the laboratory supervisor/manager before release was unavailable, as the LRFs examined in this study did not request this information.

In general, none of the DH or BTS forms examined in this study met all of the IFCC-WG QIs.^19^ Considering the frequency of omission of very vital information on both departments’ LRFs, including physician contact details, laboratory details, and test request, we suggest that both LRFs be redesigned to meet international standards.

### Limitations

One of the major limitation of this study is that the opinion of the healthcare workers involved in completing the LRFs was not sought. This would have given more insight into the reasons for the incomplete items. Another limitation of this study is its design and the differences in the two LRFs. The DH form has a total of 12 items, whereas the BTS form has 18 items. Hence, comparing the quality and completion of the two forms for the different sections should be interpreted with caution in the light of these design variations. In addition, we did not classify the LRFs in terms of inpatient or outpatient, routine or emergency service. Hence, the impact of the missing information on care of critical patients could not be assessed.

### Recommendations

This study demonstrates that the currently-used LRF for both DH and BTS should be reviewed and redesigned. The redesign should include: the physician’s phone number, time of the sample collection, time of the request, signature of the laboratory supervisor (to validate results) and the biological reference range interval in line with ISO 15189 requirements and standards.^27^ Biological reference ranges serve as guidance for the proper interpretation of laboratory test results. There is a need to develop a laboratory quality manual, guidelines and standard operating procedures, especially for sample rejection practices, as well as details on utilisation and completion of LRFs. Basic components of laboratory processing with an emphasis on the pre-analytical stage of laboratory work flow should be prominent in the orientation training of all new house officers, residents and other users of laboratory services. There is a need to revive and sustain joint physician-laboratory conferences and review meetings to share knowledge, strengthen communication and foster feedback for quality improvement. Periodic comprehensive laboratory audits with an emphasis on LRF evaluation could be beneficial in comparing baseline information with post-evaluation data for continuous quality improvement efforts. Extension of similar assessment of the LRF currently being used in other departments at Aminu Kano Teaching Hospital (Microbiology, Chemical Pathology and Histopathology) could also create an opportunity for improvement in the quality of laboratory outputs and, ultimately, on patient care.

### Conclusion

Overall completion of LRFs submitted to DH was higher compared with those submitted to BTS; however, completion of BTS LRFs was higher when assessed according to QIs. This study highlights the level of incompleteness of routinely-submitted LRFs and points out certain expected and vital pieces of information that were completely missing. This underscores the need to redesign the LRF, provide capacity building, strengthen communication between laboratory staff and physicians and enforce specimen rejection practices.

## Trustworthiness

The findings reported in this article reflect the outcome of work done on LRFs by our research team members who participated in the research design and excusion, collection, analysis of data and report writing.

### Reliability

The findings of the research presented in this report were based on a review of LRFs submitted routinely to the DH and BTS of Aminu Kano Teaching Hospital in Kano, Nigeria. Based on the study design, the findings are specific and limited to Amino Kano Teaching Hospital, Nigeria.

### Validity

The findings, outcomes and recommendations from this study may be of benefit to developing countries, such as Nigeria. In addition to the percentage performance reported, data were also subjected to four composite QIs to evaluate the most important QIs expected on LRFs. Importantly, the findings and outcomes of this study will form a baseline for comparison in future and the practical recommendations offered will help Aminu Kano Teaching Hospital to make informed decisions about re-designing the assessed LRFs and to stimulate review of other LRFs in other sections of the hospital.
